# Green Tea Polyphenol (-)-Epigallocatechin-3-Gallate (EGCG): A Time for a New Player in the Treatment of Respiratory Diseases?

**DOI:** 10.3390/antiox11081566

**Published:** 2022-08-13

**Authors:** Daniela Mokra, Jana Adamcakova, Juraj Mokry

**Affiliations:** 1 Department of Physiology, Jessenius Faculty of Medicine in Martin, Comenius University in Bratislava, SK-03601 Martin, Slovakia; 2 Department of Pharmacology, Jessenius Faculty of Medicine in Martin, Comenius University in Bratislava, SK-03601 Martin, Slovakia

**Keywords:** epigallocatechin-3-gallate, green tea, polyphenols, respiratory diseases, inflammation, oxidative stress

## Abstract

(-)-Epigallocatechin-3-gallate (EGCG) is a major polyphenol of green tea that possesses a wide variety of actions. EGCG acts as a strong antioxidant which effectively scavenges reactive oxygen species (ROS), inhibits pro-oxidant enzymes including NADPH oxidase, activates antioxidant systems including superoxide dismutase, catalase, or glutathione, and reduces abundant production of nitric oxide metabolites by inducible nitric oxide synthase. ECGC also exerts potent anti-inflammatory, anti-fibrotic, pro-apoptotic, anti-tumorous, and metabolic effects via modulation of a variety of intracellular signaling cascades. Based on this knowledge, the use of EGCG could be of benefit in respiratory diseases with acute or chronic inflammatory, oxidative, and fibrotizing processes in their pathogenesis. This article reviews current information on the biological effects of EGCG in those respiratory diseases or animal models in which EGCG has been administered, i.e., acute respiratory distress syndrome, respiratory infections, COVID-19, bronchial asthma, chronic obstructive pulmonary disease, lung fibrosis, silicosis, lung cancer, pulmonary hypertension, and lung embolism, and critically discusses effectiveness of EGCG administration in these respiratory disorders. For this review, articles in English language from the PubMed database were used.

## 1. Introduction

Recently published data from Global Burden of Disease, Injuries, and Risk Factors Study (GBD) 2017 analyzed by a group of GBD Chronic Respiratory Disease Collaborators revealed that, in 2017, 544.9 million people worldwide suffered from a chronic respiratory disease, representing an increase of 39.8% compared with the year 1990 [1]. Furthermore, chronic respiratory diseases were found to be the third leading cause of death in 2017 among all deaths (3,914,196 deaths due to respiratory diseases in 2017, an increase of 18.0% since 1990), just behind cardiovascular diseases and cancer. Total disability-adjusted life-years (DALYs) increased by 13.3%. In the European Union (EU), 339,000 deaths were reported in 2016, equivalent to 7.5% of all deaths (standardized death rate for respiratory system diseases was 74.9 deaths per 100,000 inhabitants in 2016 in the EU). However, this proportion of deaths varies considerably in various countries, as described in most recent Eurostat data from 2018 [2]. These findings confirm that chronic respiratory diseases are very common, and they are associated with substantial morbidity and mortality [3]. Furthermore, the total costs of respiratory disease prevention and therapy (direct, indirect, and monetized value of DALYs) were estimated at EUR 379.6 billion in 2011, suggesting further increase even 10 years later [4,5]. Of course, deaths due to respiratory causes significantly increased in the years 2020–2022 due to the ongoing pandemic of the coronavirus disease COVID-19; however, these data have not been made completely available. In any case, the rising trend in the incidence of respiratory diseases compels researchers to seek new approaches that may alleviate the course of these disorders, which seriously limit the quality of life.

Respiratory diseases such as acute respiratory distress syndrome (ARDS)/acute lung injury (ALI) [6,7,8,9], respiratory infections including COVID-19 [10,11,12], bronchial asthma [13,14], chronic obstructive pulmonary disease (COPD) [13,15,16], pulmonary fibrosis [17,18], silicosis [19,20], lung cancer [21,22], pulmonary hypertension [23], and lung embolism [24] are at least partially associated with inflammation with abundant accumulation and activation of inflammatory cells in the airways and/or lung parenchyma, e.g., neutrophils in ALI [8] and eosinophils/neutrophils in bronchial asthma [25]. The inflammation is associated with overproduction of various bioactive substances including pro-inflammatory cytokines such as tumor necrosis factor (TNF)α, interleukin (IL)-1β, IL-6, IL-8, etc., reactive oxygen species (ROS) such as hydroxyl radicals, peroxides, superoxide anions, etc., proteases such as neutrophil elastase, etc. Dysregulation of inflammation and oxidant/antioxidant dysbalance may subsequently progress into chronic tissue damage and fibrotizing changes [8,9,12,13,18,26,27]. This finding is extremely important, since the lung is an organ exposed to oxidative stress already under physiological conditions; therefore, the lung possesses a multi-level protective antioxidant system [27,28].

Understanding the fundamental role of inflammation and inflammation-related oxidative stress in the onset and progression of respiratory diseases has led to use of various antioxidants in the treatment [19,26,27,29,30,31]. However, the effectiveness of many existing synthetic antioxidants is not sufficient, or their administration is associated with undesirable side effects. Therefore, searching for natural-based compounds seems to be a promising approach. Among the possibilities of prevention and treatment for respiratory diseases, natural flavonoids, a wide group of polyphenolic compounds present in plants, should be considered [32,33]. For instance, a polyphenol (-)-epigallocatechin-3-gallate (EGCG) occurring in the green tea plant (*Camellia sinensis*) has demonstrated a broad spectrum of anti-inflammatory, antioxidant and anti-fibrotic effects, which may also be useful for the treatment of respiratory diseases [34,35,36,37,38,39,40,41]. This article reviews current information on the biological effects of EGCG in those respiratory diseases or animal models in which EGCG has been administered, i.e., acute respiratory distress syndrome, respiratory infections, COVID-19, bronchial asthma, chronic obstructive pulmonary disease, lung fibrosis, silicosis, lung cancer, pulmonary hypertension, and lung embolism, and critically discusses the effectiveness of EGCG administration in these respiratory disorders. For the review, articles in English language from the PubMed database were used.

## 2. Epigallocatechin-Gallate (EGCG)

**Figure 1:**
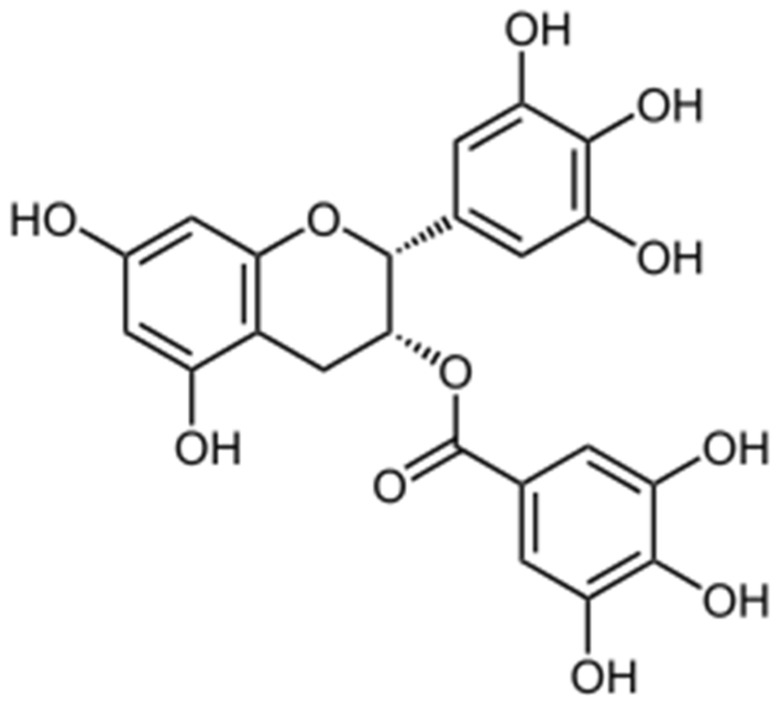
Chemical formula of EGCG [48].

The chemical composition of green tea depends on many factors, including climate, season, horticultural practices, processing, and type and age of the plant [42,43]. Green tea contains polyphenols, i.e., flavanols, flavandiols, flavonoids, and phenolic acids, which account for 30% of the dry weight of green tea leaves. Majority of the green tea polyphenols represent flavanols, commonly known as catechins, from which the most important are EGCG, (-)-epicatechin (EC), (-)-epicatechin-3-gallate (ECG), and (-)-epigallocatechin (EGC) [44]. EGCG (Figure 1) forms 50–80% of green tea catechins; therefore, its content in a cup of brewed tea is estimated to be 200–300 mg [45,46]. Plasma concentration of catechins reaches a peak value in 1–4 h after ingestion of green tea or catechin supplements and gradually lowers back to baseline value within 24 h [47].

Complex analyses of biological effects of green tea polyphenols have shown that there are rather large differences in their pharmacokinetics among the individual polyphenols [49,50]. For instance, after ingestion of 1.5 mM of tea polyphenols by healthy volunteers, plasma level of EGC elevated quickly with a short elimination half-time of 1.7 h, while EGCG was the slowest to increase, but exhibited an intermediate decrease, with an elimination half-life of 3.9 h [49]. In additional measurements, maximum plasma concentrations reached 223 ng/mL for EGC and 78 ng/mL for EGCG, with no differences in pharmacokinetic parameters between ingestion of decaffeinated green tea or pure EGCG. In the plasma, EGCG was present mostly in a free form, while EGC was mainly present in a conjugated form [50].

Further differences in tea polyphenols were observed with respect to their biological effects which are likely attributable to structural differences, particularly regarding the presence or absence of galloyl moiety [46]. Thus, tea catechins containing galloyl moiety (i.e., EGCG and ECG) exerted stronger biological activities [51]. For instance, EGCG had a potent inhibitory effect on histamine and leukotriene B4 release, while ECG and EGC showed a moderate effect and EC no effect [52]. Similarly, some of the metabolic actions of tea polyphenols may be related to lipid lowering effect of galloyl moiety, leading to delayed intestinal absorption of triacylglycerols and reduced deposition of visceral fat [53]. In addition, the presence of the galloyl moiety esterified at carbon 3 on the C ring and a presence of hydroxyl groups at carbons 3′, 4′, and 5′ on the B ring of EGCG molecule are likely attributable for the most potent antioxidant activity of EGCG in comparison to other catechins [54,55]. 

Nevertheless, the biological effects of EGCG are concentration dependent [51,56], as well. While low concentrations of EGCG (plasma levels of ≤10 µM) have demonstrated antioxidant action [57,58] and amelioration of insulin resistance [59], high concentrations of EGCG (>10 μM) may act as a pro-oxidant agent enhancing autophagy and cell death [44], and thereby may be utilized, e.g., in the treatment of tumors [56].

However, the effectiveness of EGCG is limited due to its poor pharmacokinetics and low bioavailability after oral delivery [60]. After ingestion of tea, only a small fraction of catechins is systemically available, i.e., can be absorbed from the intestine and consequently present in the blood and tissues. This is presumably caused by low stability in the digestive system due to extreme pH conditions and action of digestive enzymes, and by the limited membrane permeability across the intestinal wall based on passive diffusion without specific receptors carrying EGCG into the intestinal cells [61,62]. The oral bioavailability of EGCG is also reduced by food intake; thus, the maximum systemic absorption was found when EGCG capsules were taken on an empty stomach or taken with water [63].

## 3. Antioxidant Mechanisms of EGCG

**Figure 2:**
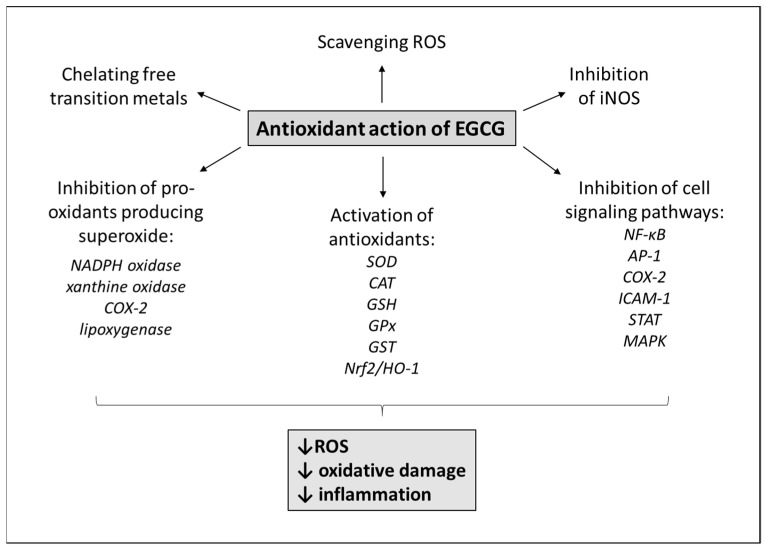
Antioxidant action of EGCG. Abbreviations: *AP-1*: activator protein 1, *CAT*: catalase, *COX-2*: cyclooxygenase-2, EGCG: epigallocatechin-gallate, *GPx*: glutathione peroxidase, *GSH*: glutathione, *GST*: glutathione-S-transferase, iNOS: inducible nitric oxide synthase, *HO-1*: heme oxygenase-1, *ICAM-1*: intercellular adhesion molecule-1, *MAPK*: mitogen-activated protein kinase, *NADPH*: nicotinamide adenine dinucleotide phosphate, *NF-κB*: nuclear factor kappa-B, *Nrf2*: nuclear factor erythroid-derived 2-like 2, ROS: reactive oxygen species, *SOD*: superoxide dismutase, *STAT*: signal transducer and activator of transcription.

As mentioned before, a relatively large area of the lungs is exposed to huge amounts of ROS in inhaled air or produced by activated immune cells in the lungs [64]. ROS are created by metabolizing organelles, especially by mitochondria, peroxisomes and endoplasmic reticulum [65], and under normal conditions, small concentrations of produced ROS are eliminated by antioxidant systems [66]. However, a shift towards oxidative stress triggers various signaling pathways which stimulate both inflammation and carcinogenesis, such as transcription factors nuclear factor (NF)-κB, activator protein (AP)-1, signal transducer and activator of transcription (STAT)3, protein kinases such as mitogen-activated protein kinase (MAPK) or c-Jun NH2-terminal kinase (JNK), cell adhesion molecules such as intercellular adhesion molecule (ICAM), cyclooxygenase (COX)-2, and many others [67,68,69,70]. The majority of the mentioned pathways can be modulated by EGCG, which thereby alleviates inflammation and cell proliferation [71,72,73]. Nevertheless, exceptional property of flavonoids including EGCG is their complex antioxidant action supplied by several mechanisms [74,75] (Figure 2).

Direct antioxidant action of EGCG can be mediated by chelating free transition metals (iron, copper), which amplify the ROS formation [76,77]. Action of EGCG as a radical scavenger is related to its one-electron reduction potential, an ability to function as hydrogen or electron donor [78]. This means that antioxidants react with free radicals by two mechanisms: they can perform hydrogen atom transfer reaction (HAT), where the free radical removes one hydrogen atom from antioxidant, and the antioxidant itself becomes a radical, or the antioxidants perform the single electron transfer reaction (SET), where the antioxidant provides an electron to the free radical and itself then becomes a radical cation, where both reactions involve hydroxyl groups [79]. In particular, the presence of ortho-dihydroxyl group on the B and D rings and a galloyl moiety on the 3 position increases the ability of EGCG to scavenge free radicals (mainly superoxide anions, hydroxyl radicals, and 1,1-diphenyl-3-picrylhydrazyl radicals) in comparison to other catechins [54,55]. 

Another important mechanism is the inhibition of pro-oxidant enzymes producing superoxide anions, such as NADPH oxidase, xanthine oxidase, COX-2, lipoxygenase, mitochondrial succinoxidase, microsomal monooxygenase, etc. [74,80,81]. Nicotinamide adenine dinucleotide phosphate (NADPH) oxidase (NOX) is a major source of ROS in various types of cells including neutrophils and vascular endothelial cells. NOX is a membrane-associated enzyme using NADPH as an electron donor for reduction of oxygen to superoxide radical anion. NOX has seven isoforms (NOX1-5 and DUOX1-2) that exert distinct actions [82,83]. For instance, NOX2 is expressed in phagocytes, where the produced superoxide serves for the destruction of microorganisms [84]. However, NOX2 is also expressed in other cells, including endothelial cells, where it stimulates cell proliferation, and higher activity of NOX2 is associated with atherosclerosis, hypertension and pulmonary arterial hypertension [82]. NOX4 is produced, e.g., in vascular endothelium and smooth muscle cells where the hydrogen peroxide production is essential for cell proliferation and differentiation, but ROS overexpression contributes to atherosclerosis [85,86]. Moreover, NOX4 was found to contribute to epithelial cell death in the lung fibrosis [87]. EGCG effectively suppressed NADPH oxidase and ROS production, e.g., in TNFα-induced inflammation [88], as well as in COVID-19 [89]. Another pro-oxidant enzyme, xanthine oxidase, is responsible for catabolism of purines and their conversion into uric acid; however, higher activity associated with ROS overproduction was also found in sepsis [90] and models of ALI [91]. EGCG effectively inhibits the activity of xanthine oxidase [92]. COX-2 is a fundamental enzyme in fatty acid metabolism. Moreover, COX-2 is upregulated in inflammatory situations and cancer and EGCG inhibited its expression in activated macrophages [93], as well as in premalignant and malignant conditions [94,95].

Moreover, flavonoids alleviate oxidative stress induced by nitric oxide (NO), which in normal amounts contributes to many physiological processes including vasodilation. However, high concentrations of NO produced by inducible NO synthase (iNOS) act as a pro-inflammatory mediator. In addition, the production of NO under oxidative stress secondarily generates a production of potent oxidizing agents, i.e., reactive nitrogen species (RNS) such as peroxinitrite, which is formed in the reaction of NO with superoxide [96]. EGCG was shown to inhibit iNOS activity [97,98], enhance the activity of constitutive NOS [99], and enhance the bioavailability of normal NO.

Indirect antioxidant action of flavonoids is also related to inhibition of redox-sensitive transcription factors, such as NF-κB or AP-1, which leads to suppression of inflammation and, thereby, reduced production of ROS by activated immune cells [100]. 

Antioxidant action of flavonoids is also related to induction of phase II detoxifying antioxidant enzymes, such as glutathione S-transferase (GST), NAD(P)H-quinone oxidoreductase, uridine diphospho(UDP)-glucuronosyl transferase or superoxide dismutase (SOD), which are responsible for elimination/deactivation of electrophilic forms of carcinogens or inactivation of ROS [51,77,101]. Glutathione (γ-glutamylcysteinylglycine, GSH) is the most abundant non-protein thiol protecting from oxidative stress; however, GSH participates in detoxification of xenobiotics and regulates many processes including cell proliferation, apoptosis, immune functions, and fibrogenesis. GSH is synthetized in two steps. In the first step, γ-glutamylcysteine is formed from sulfur amino acid precursor cysteine and glutamate what is catalyzed by glutamate-cysteine ligase (GCL) consisting of catalytic and modifier subunits (GCLC and GCLM). The second step of synthesis from γ-glutamylcysteine and glycine to γ-glutamylcysteinylglycine is catalyzed by GSH synthase (GS) [102]. GSH exists in two forms: thiol-reduced (GSH) and disulfied-oxidized (GSSH). Antioxidant action of GSH is exerted in glutathione peroxidase (GPx)-catalyzed reactions where hydrogen peroxide and lipid peroxide are reduced and GSH is oxidized to GSSG. GSSG is reduced back to GSH by GSSG reductase (GR) at the expense of NADPH [103]. However, severe oxidative stress depletes cellular pools of GSH [103]. The antioxidant function of GSH is particularly important in mitochondria [104]. As many transcription factors and signaling molecules have cysteine residues that can be oxidized, ROS- and/or RNS-mediated regulation of protein function and cell signaling may be modulated by GSH system. Thus, in addition to keeping redox balance GST regulates many physiological reactions including immune functions, fibrogenesis, cell growth and death [102]. EGCG clearly showed a potential to enhance activity of the mentioned antioxidant enzymes in models of various respiratory diseases [36,77,97,105,106,107,108].

The key role in regulating induction of phase II detoxifying or antioxidant enzymes is played by a redox-sensitive transcription factor, nuclear factor erythroid 2 p45 (NF-E2)-related factor (Nrf2), which mediates their transcriptional activation through the interaction of Nrf2 with the antioxidant-response element (ARE) or the electrophile-responsive element (EpRE) [101,109]. In addition to inducing phase II detoxifying enzymes [109], Nrf2 acts in de novo synthesis of antioxidant enzymes protecting from cytotoxicity caused by oxidative stress [110], or pro-inflammatory mediators [111]. Another antioxidant system activated by Nrf2 is heme oxygenase (HO)-1 [112]. HO-1 is an enzyme responsible for degradation of heme to carbon monoxide (CO), free iron and biliverdin-IXα. Since biliverdin-IXα is converted to bilirubin-IXα, an endogenous scavenger of radicals, iron is sequestered into ferritin which together with CO exert antioxidant and anti-apoptotic effects [88,113]. EGCG induced expression of both Nrf2 and HO-1, which resulted in antioxidant and anti-inflammatory effects [34,107,114,115].

## 4. Effects of EGCG in Non-Respiratory Diseases

A variety of actions of EGCG have been described, particularly in the relation to cancer [116,117,118,119,120,121,122]; however, an improvement associated with delivery of EGCG has been also observed in many other disorders such as brain aging [123,124], neurological diseases including Parkinson’s and Alzheimer’s diseases [125,126], cardiovascular diseases [127,128,129], and metabolic diseases including obesity [130,131] and diabetes mellitus [132,133]. 

## 5. Effects of EGCG in Respiratory Diseases

Thanks to a wide spectrum of anti-inflammatory, antioxidant, and anti-fibrotizing effects, EGCG is also increasingly being used in the treatment of acute and chronic respiratory diseases. 

**Figure 3:**
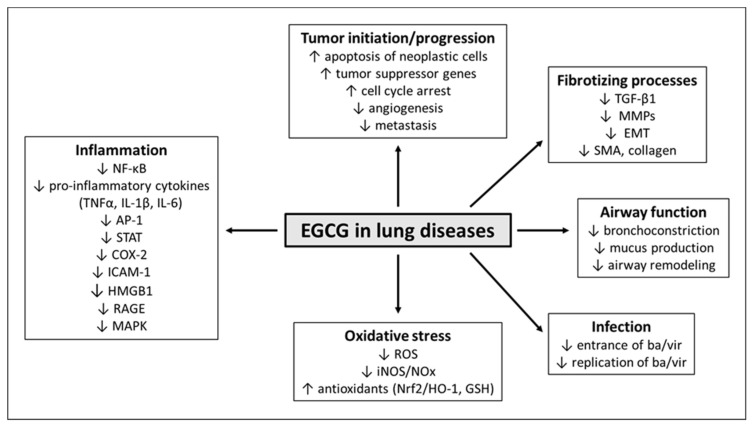
Major effects of EGCG in the lung diseases. Abbreviations: AP-1: activator protein 1, ba: bacteria, COX-2: cyclooxygenase-2, EGCG: epigallocatechin-gallate, EMT: epithelial–mesenchymal transition, GSH: glutathione, iNOS: inducible nitric oxide synthase, HMGB1: high-mobility group box 1, HO-1: heme oxygenase-1, ICAM-1: intercellular adhesion molecule, MAPK: mitogen-activated protein kinase, MMPs: matrix metalloproteinases, NF-κB: nuclear factor kappa-B, NOx: nitric oxide metabolites, Nrf2: nuclear factor erythroid-derived 2-like 2, RAGE: receptor for advanced glycation end products, ROS: reactive oxygen species, SMA: smooth muscle actin, STAT: signal transducer and activator of transcription, TGF-β1: transforming growth factor-beta1, TNFα: tumor necrosis factor alpha, IL-1β: interleukin-1beta, vi: viruses.

The main mechanisms of EGCG in respiratory diseases are displayed in Figure 3.

A review of the major targets of EGCG action in the lung is provided in Table 1.

### 5.1. EGCG in ALI

EGCG has been successfully used in animal models of ALI resembling clinical ARDS (Table 2). These disorders originate from direct (pulmonary) causes such as pneumonia, near drowning, or inhalation of toxic gases, or from indirect (extrapulmonary) causes such as sepsis, severe trauma, or acute pancreatitis [8]. In response to lung injury, there are complex interactions between the circulating polymorphonuclears, particularly neutrophils, and the vascular endothelium. Activated neutrophils play a crucial role in overproduction of ROS, as well [30]. 

In A549 cells and human pulmonary alveolar epithelial cells as well as in the lung of mice, TNFα increased expression of ICAM-1 contributing to the recruitment of polymorphonuclears to the inflammatory site; however, pretreatment with EGCG decreased ICAM-1 expression and the counts of neutrophils and eosinophils in the bronchoalveolar lavage fluid (BALF). EGCG also inhibited TNFα-induced NADPH oxidase activation and ROS generation, MAPK phosphorylation, and phosphorylation of STAT3 and activating transcription factor (ATF)2. In addition, EGCG induced expressions of heme oxygenase (HO)-1, known for its antioxidant action, and suppressors of cytokine signaling (SOCS)-3 proteins, negatively regulating cytokine signaling. These results indicate that HO-1 or SOCS-3 suppresses the TNFα signaling, not only by decreasing expression of adhesion molecules, but also by reducing ROS production and STAT-3 and ATF2 activation [148].

In pulmonary inflammation induced by intratracheal (i.t.) lipopolysaccharide (LPS) in mice, pretreatment with EGCG given 1 h before LPS alleviated the lung injury, decreased total cell, neutrophil, and macrophage counts in the lung, reduced a lung edema, decreased activities of myeloperoxidase (MPO) and protein kinase Cα, and lowered levels of pro-inflammatory cytokines TNFα, IL-1β and IL-6 [39]. A similar effect of EGCG on i.t. LPS-induced lung damage and inflammation was observed in another study where, in addition to the above-mentioned changes, EGCG modulated the polarization of macrophages towards an anti-inflammatory phenotype M2, including an increase in expression of mediators supporting M2 phenotype such as Krüppel-like factor (KLF)4, arginase gene (Arg)1 and macrophage secretory protein ym1 [98]. Moreover, EGCG mitigated oxidative damage, which was demonstrated as a decline in oxidation markers, 8-hydroxy-2-deoxyguanosine (8-OHdG) and nitrotyrosine, and enhanced the regeneration capacity of the lung, which was confirmed by an increase in expression of markers of cell proliferation such as nuclear antigen Ki67 and proliferating cell nuclear antigen (PCNA), and angiopoietin-1 [98]. 

In systemic inflammation induced by intraperitoneal (i.p.) LPS, pretreatment with EGCG decreased arterial partial pressure of carbon dioxide (PaCO_2_) and increased arterial partial pressure of oxygen (PaO_2_) and pH demonstrating improved lung function and acid-base balance, decreased formation of lung edema, mitigated a severity of histopathological changes, especially for infiltration with inflammatory cells and hemorrhage, reduced MPO activity and expression of TNFα, IL-1β and IL-6 in the lung, serum and BALF, alleviated expression of toll-like receptor (TLR)4, myeloid differentiation primary response (MyD)88 protein, TIR-domain-containing adapter-inducing interferon-β (TRIF), and transcription factor p-p65 in the lung, and elevated expression of nuclear factor of kappa light polypeptide gene enhancer in B-cells inhibitor (IκB)-α, suggesting that the anti-inflammatory action may be related to suppression of activation of TLR4-dependent NF-κB signaling pathway [136]. In fluoride-induced oxidative stress mediated lung injury in rats, pretreatment with EGCG lowered inflammatory cytokines such as TNFα, IL-1β, IL-6, and cytokine induced neutrophil chemoattractant (CINC)-3, decreased MPO as a marker of neutrophil accumulation, and lung edema, reduced oxidative stress (expressed by a decrease in superoxide radicals, hydroxyl radicals and hydrogen peroxide and lower levels of malondialdehyde (MDA) and increased levels of both non-enzymatic antioxidants (GSH and vitamin E) and enzymatic antioxidants (SOD, catalase, GPx, GR, GST), while the antioxidant action was attributed to activation of the Nrf2/Keap1 pathway [150]. 

### 5.2. EGCG in Bacterial and Viral Respiratory Infections

Infections of the upper and lower respiratory tract may be caused by a broad spectrum of bacteria, viruses, and fungi. To the most frequent bacterial species belong, for instance, *Streptococcus pneumoniae*, *Staphylococcus aureus*, *Haemophillus pneumoniae*, *Klebsiella pneumoniae*, *Mycoplasma pneumonia*, *Mycobacterium tuberculosis* or *Pseudomonas aeruginosa*, viral infections may be caused by *influenza* virus or respiratory syncytial virus [10,11], and nowadays also by severe acute respiratory syndrome coronavirus 2 (SARS-CoV-2). Effects of EGCG in several models of respiratory infections are provided in Table 2. 

The anti-bacterial properties of EGCG have been demonstrated in several animal models of pneumonia induced by bacteria or viruses. In mice with *Pseudomonas aeruginosa*-induced pneumonia, EGCG alleviated lung damage, reduced pathological signs of injury and pulmonary edema, decreased *Pseudomonas aeruginosa* load and virulence factors, suppressed expression of TNFα, IL-1β, IL-6, and IL-17 in the lung and simultaneously enhanced expression of anti-inflammatory cytokines IL-4 and IL-10 [41]. Similarly, microencapsulated EGCG given for 5 days per week for 6 weeks by aerosolization using low-density porous trehalose microspheres as a delivery vehicle led to resolution of inflammation in the *Mycobacterium tuberculosis*-infected lung by enhancing the autophagy and reduction in bacterial burden [150]. 

Anti-viral activity of EGCG against *influenza A* virus was tested in BALB/c mice in vivo as well as in canine kidney cells in vitro [151]. In mice, EGCG was given in three different doses for 5 days, and *influenza A* infection was induced by intranasal inoculation with FluA (FM1 strain) on the third day of EGCG treatment. Oral administration of EGCG (40 mg/kg/d) enhanced the survival rate, decreased the mean virus yields and alleviated pneumonia in the lung of mice while in vitro measurements inhibited *influenza A* replication in a concentration-dependent manner and suppression of *influenza A*-induced increase in ROS level [151]. The anti-viral, anti-bacterial and anti-fungal properties of EGCG were corroborated in detail in an excellent review article by Steinmann et al. [153].

#### EGCG in COVID-19

In light of the ongoing pandemic of COVID-19 and searching for novel therapeutic approaches, the effects of EGCG have been recently published in several articles [154,155,156,157,158]. EGCG may suppress SARS-CoV-2 infection via activation of Nrf2, the transcription factor regulating many processes, including anti-viral response [159]. Fundamental factors for entry of coronavirus into the cell include angiotensin-converting enzyme 2 (ACE2), a cell receptor for SARS-CoV-2 cell entry, and serine protease TMPRSS2 for spike protein priming [160]. EGCG similarly to other Nrf2-activators blocked infection of SARS-CoV-2 and new variants by inhibiting spike binding to ACE2 receptor [40,161]. EGCG also reduced a replication of SARS-CoV-2 via inhibition of the main protease (3CLpro) of the virus, which contributes to viral replication and gene expression of viral proteins, in both in vitro studies [162,163] and in vivo murine model [152]. In addition, EGCG may reduce SARS-CoV-2 replication by suppressing generation of ROS in mitochondria and oxidative burst associated with neutrophil extracellular traps (NETs), which stimulate viral replication in the cells [12]. However, SARS-CoV-2-induced oxidative stress also promotes lung tissue damage through several mechanisms [154]. In SARS-CoV infection, antioxidant mechanisms are suppressed as demonstrated by decreases in total antioxidant capacity (TAC), GSH [164,165], SOD [12], or selenoprotein P [166]. On the other hand, generation of ROS massively increases because of virus-stimulated activity of ROS-generating enzymes including NOX, excessive accumulation and activation of neutrophils in the lung [167] associated with increased neutrophil-to-lymphocyte ratio in the blood [168], abundant formation of NETs [169,170], and increased activity of NOX4, xanthine oxidase/reductase or endothelial/inducible nitric oxide synthases because of lung tissue injury-induced hypoxia [171]. Increased oxidative stress in COVID-19 was demonstrated in several studies as an increase in total oxidant status (TOS) and oxidative stress index [164,165], or elevated levels of thiobarbituric acid reactive substances (TBARS), a marker of lipid peroxidation, and F2-isoprostane, a marker of oxidant damage [172], whereas the increase in oxidative stress and decrease in antioxidant levels in COVID-19-infected patients were associated with worsening of disease [164,165]. EGCG, via its broad antioxidant action, may reduce the oxidative stress by direct scavenging of various ROS, reducing NOX [89], and by inducing antioxidant and detoxifying enzymes, such as HO-1, quinone reductase, glutamate cysteine ligase, GST, thioredoxin reductase, GR, SOD, catalase and GPx [71,101]. It is also presumed [154] that EGCG may protect mitochondria [173] from SARS-CoV-2-induced alteration in bioenergetics and dysfunction [174] and to diminish SARS-CoV-2-induced endoplasmic reticulum stress [175]. EGCG may inhibit a life cycle of SARS-CoV-2 by suppression of endoplasmic reticulum-resident glucose-regulated protein (GRP)78 activity and expression [176]. Moreover, by downregulation of TLR4 and NF-κB, EGCG may reduce a cytokine storm in COVID-19 [154].

EGCG may also alleviate the COVID-19-associated complications, such as sepsis, thrombosis, or lung fibrosis [154]. EGCG directly or via inhibiting STAT1 activation reduces high-mobility group box (HMGB)1, a redox-sensitive pro-inflammatory nuclear protein mediating sepsis [177,178]. By inhibiting cytoplasmic Ca^2+^ increase, EGCG modulates the activity of platelets [179] and decreasing tissue factors prevents thrombosis [180]. General mechanisms of how EGCG may mitigate lung fibrosis are described in the following section. The use of EGCG may be of exceptional benefit in COVID-19 associated with diabetes mellitus. Hyperglycemia upregulates receptor for advanced glycation end products (RAGE), a major mediator of pulmonary inflammatory responses including those in COVID-19, and RAGE ligands such as sepsis-associated HMGB1 [181], and this activates NF-κB via a positive regulation loop [182]. Thus, hyperglycemia increases RAGE expression and HMGB1 levels, both leading to amplification of a SARS-CoV-2/HMGB1/RAGE axis [181]. Previous findings that EGCG dose-dependently downregulated RAGE and increased a soluble RAGE competing with RAGE in patients with type II diabetes [183] indicate that EGCG may decrease the mortality of COVID-19 patients with diabetes comorbidity.

### 5.3. EGCG in Bronchial Asthma

Bronchial asthma is a heterogenic disorder presenting in several endotypes with distinct pathophysiological backgrounds and phenotypes and with special clinical characteristics. Therefore, chronic airway inflammation leading to airway remodeling, mucus hypersecretion metaplasia and hyperplasia of goblet cells, and hypertrophy and hyperplasia of airway smooth muscle may, in the allergic or eosinophilic endotype of asthma, result from allergen sensitization (pollen, house dust mite, etc.) or may be related to frequent respiratory infections, obesity, air pollution or smoking in the non-eosinophilic endotype [25].

EGCG can also be of benefit in bronchial asthma, as has been demonstrated in various models of this disease (Table 3).

For instance, pretreatment with EGCG before ovalbumin (OVA) challenge significantly reduced bronchoconstriction, decreased inflammatory cell recruitment, free radical lung injury, and release of proinflammatory molecules in BALF, and enhanced endothelial NO synthase (eNOS) activity [99]. In a murine model of allergic asthma, EGCG decreased mucus production, mucin (MUC)5B expression, p38 MAPK expression, and matrix metalloproteinase (MMP)-9 expression, which was also confirmed in nasal epithelial cells of patients with allergic rhinitis [184]. In OVA-challenged asthmatic mice, EGCG lowered the number of total leukocytes, as well as counts of macrophages, eosinophils and neutrophils, in the BALF, and decreased epithelial–mesenchymal transition (EMT) under the influence of transforming growth factor (TGF)-β1 and PI3K/Akt signaling pathway, which suggests the ability of EGCG to prevent airway remodeling [138]. In later experiments performed by these authors, EGCG given at two different doses 1 h after each OVA challenge decreased OVA-induced hyperreactivity and OVA-specific immunoglobulin (Ig)E in serum, alleviated airway inflammation as expressed by decreased eosinophils, elevated concentrations of anti-inflammatory cytokine IL-10, and increased the number of CD4+CD25+Foxp3+Treg cells and expression of Foxp3 mRNA in the lung tissue regulating T-cell (Treg/Th17) balance [185]. In mice with OVA-induced asthma, EGCG treatment significantly reduced asthma symptoms and decreased numbers of eosinophils and neutrophils in the BALF, decreased IL-2, IL-6, and TNFα, increased IL-10 concentrations, diminished percentage of Th17 cells, increased percentage of Treg cells, and decreased expressions of TGF-β1 and phosphorylated (p)-Smad2/3 [37].

In an OVA-induced model of allergic asthma associated with obesity, EGCG reduced total cells and eosinophils in the lung, normalized levels of TNFα, IL-4, IL-5, and eotaxin in BALF, expressions of iNOS and NO metabolites (NOx), and levels of ROS and SOD in the lung tissue; however, EGCG had no significant effect on the mentioned parameters in lean animals [97].

In another model of asthma induced by toluene diisocyanate-inhalation, administration of EGCG suppressed asthmatic reaction, decreased the number of inflammatory cells in BALF and their infiltration into the airways, decreased the expression of MMP-9 mRNA and protein in the lung tissue, and diminished ROS, TNFα, and IL-5 concentrations in BALF [186].

In fine particulate matter-induced asthma model in rats, EGCG mitigated lung injury and inflammatory cell infiltration, decreased bronchial wall and bronchial smooth muscle thickness, and reduced the expression of HMGB1 and RAGE mRNA and protein, contributing to inflammatory cascade in asthma, while more obvious results were observed for higher doses of EGCG [38]. 

**Table 3:** EGCG in the treatment of bronchial asthma, COPD, lung fibrosis, silicosis, and lung cancer (animal models). For more details, see the text.

Animal Model	Species	EGCG Dose/Way of Delivery	Major Findings	Study
OVA-induced model of bronchial asthma	Guinea pigs	EGCG (25 mg/kg s.c.) given 20 min prior to OVA challenge	↓ bronchoconstriction, ↓ inflammation, ↓ lung injury; ↑ eNOS activity	[99]
OVA-induced model of bronchial asthma	Balb/c mice	EGCG (0.5 mg/mL in drinking water) given for 8 weeks, started 1 h after the 1st OVA challenge	↓ cell counts in BALF, ↓ inflammation and EMT	[138]
OVA-induced model of bronchial asthma	Balb/c mice	EGCG (10 or 20 mg/kg/d i.v.) given 3 d after OVA sensibilization and challenge	↓ bronchoconstriction and inflammation, ↓ TGF-β1 and phosphorylated (p)-Smad2/3	[37]
OVA-induced model of bronchial asthma	Balb/c mice	EGCG (5 or 50 mg/kg i.p.) given 1 h before each OVA challenge, for 30 d	↓ bronchoconstriction and inflammation	[185]
Obesity-associated OVA-induced asthma	C57BL/6 mice	EGCG (10 mg/kg/day, gavage, for 2 weeks) given simultaneously with OVA sensitization	↓ inflammation, ↓ ROS, ↑ SOD, ↓ iNOS and NOx	[97]
Toluene diisocyanate (TDI)-inhalation induced model of bronchial asthma	Balb/c mice	EGCG (0.3% in drinking water) given for 10 d from last sensitization to 2 days after first challenge	↓ bronchoconstriction, ↓ cells in BALF, ↓ MMP-9 in the lung, ↓ ROS, TNFα, and IL-5 in BALF	[186]
Fine particulate matter 2.5 (PM_2.5_)-induced model of bronchial asthma	Sprague-Dawley rats	EGCG (10 or 50 mg/kg i.p.) given 1 h before 1st atomization of PM_2.5_ (10 mg/kg, by i.t. atomization done 4-times every other day)	↓ lung injury and inflammation, ↓ bronchial smooth muscle thickness, ↓ HMGB1 and RAGE	[38]
House dust mite (HDM)-induced asthma	C57BL/6 mice	EGCG (50 mg/kg i.p.) given 1 h before HDM challenge	↓ tissue injury, ↓ inflammation, ↓ mucus production, ↓ collagen deposition, ↓ M2 macrophages in the lung	[187]
Cigarette smoke (CS)-induced model of COPD	Sprague-Dawley rats	EGCG (50 mg/kg) given by oral gavage every other day during 56 d of cigarette smoke exposure	↓ markers of oxidative stress and neutrophil inflammation, ↑ SOD, catalase, GST, ↓ mucus, ↓ airway remodeling	[36]
Bleomycin-induced lung fibrosis	Wistar rats	EGCG (20 mg/kg i.p.) given for 28 d, started 6 h after bleomycin (6.5 U/kg i.t.) instillation	↓ lung injury, inflammation, and fibrosis, ↓ ROS, ↑ antioxidants	[34,35,188,189]
Irradiation-induced pulmonary fibrosis	Sprague-Dawley rats	EGCG (25 mg/kg i.p.) given for 30 d, started after (60)Co irradiation (22 Gy)	↓ mortality, ↓ lung injury, inflammation, and fibrosis	[106]
Cyclophosphamide-induced pulmonary fibrosis	Wistar rats	Green tea extract (150 mg/kg i.g.) given for 14 d, before cyclophosphamide (150 mg/kg i.p.) administration in 2 consecutive days	↓ oxidative stress, inflammation, and fibrosis	[114]
Paraquat-induced pulmonary fibrosis	Sprague-Dawley rats	Green tea extract (1% i.g.), after paraquat (0.3 mg/kg i.t.) instillation	↓ oxidative stress and ET-1	[190]
Particulate silica-induced lung fibrosis	Sprague-Dawley rats	EGCG (50 mg/kg), PBCA-NPs (150 mg/kg) or their combination, given daily by gavage for 28 d, started 2 d after silicosis modeling (SiO_2_ 50 mg/mL, 1 mL i.t.)	↓ fibrosis, restored body weight	[191]
CS-induced model of bronchial cells dysplasia	Sprague-Dawley rats	EGCG (0.3%) in drinking water, given paralelly with inhalation of CS for 4, 8, 12 or 16 weeks	↓ benzopyrene-DNA adducts, ↓ precancerous lesions of bronchial cells	[192]

Abbreviations: ALI: acute lung injury, BALF: bronchoalveolar lavage fluid, CFU: colony forming units, CS: cigarette smoke, EMT: epithelial–mesenchymal transition, eNOS: endothelial nitric oxide synthase, ET-1: endothelin-1, GST: glutathione, HMGB: high-mobility group box, IL-5: interleukin-5, iNOS: inducible nitric oxide synthase, LPS: lipopolysaccharide, i.g.: intragastric administration, i.n.: intranasal administration, i.p.: intraperitoneal administration, i.t.: intratracheal administration, MMP: matrix metalloproteinase, NOx: nitric oxide metabolites, p.o.: peroral administration, PFU: plaque-forming units, PBCA-NPs: EGCG-encapsulated poly(butyl-2-cyanoacrylate) nanoparticles, RAGE: receptor for advanced glycation end products, ROS: reactive oxygen species, SOD: superoxide dismutase, TGF-β1: transforming growth factor-beta1, TNFα: tumor necrosis factor alpha, ↓: decrease, ↑: increase.

In a house dust mite (HDM)-induced asthma model, EGCG decreased tissue injury, inflammation, mucus production and collagen deposition, and alleviated HDM-induced M2 macrophage infiltration in the lung, probably via suppressing hypoxia-inducible factor (HIF-1)α/vascular endothelial growth factor (VEGF)A-mediated M2 skewing of macrophages [187]. 

### 5.4. EGCG in COPD

COPD is a group of respiratory conditions covering lung emphysema and chronic bronchitis, characterized by breathlessness, cough, recurrent respiratory infections, and airflow limitation, with lower values of the ratio between the first second of forced expiration (FEV1) and the full forced vital capacity (FVC), a vital capacity marker, than 0.7 [193]. The most important risk factor is tobacco smoking; however, COPD may be also caused by indoor and outdoor pollution including biomass smoke, occupational exposure to irritants, e.g., biological dust, deficiency of α1-antitrypsin, etc. [194].

EGCG could also be beneficial in COPD; however, only a small number of studies have been published to date. In a cigarette smoke (CS)-induced COPD model in rats, EGCG decreased 8-isoprostane and advanced oxidation protein products (AOPP), markers of oxidative stress, and reversed activities of antioxidant enzymes (SOD, catalase, GST). In addition, EGCG lowered CINC-1, resembling human IL-8, and monocyte chemotactic protein-1 (MCP-1), markers of neutrophil-mediated inflammation, and decreased neutrophil infiltration in the lung. EGCG reduced several goblet cells and inhibited a secretion of mucus likely via inhibition of epidermal growth factor receptor (EGFR) and, finally, reduced small airway remodeling by decreasing collagen deposition [36] (Table 3). One of the more recent in vitro studies showed that EGCG has the potential to decrease CS-induced oxidative changes, lipid peroxidation and inflammation in human bronchial epithelial cells as demonstrated by decreased production of ROS and 4-hydroxynonenal in airway epithelial cells and inhibited activation of NF-κB and the associated pro-inflammatory cytokines [195]. A cross-sectional survey from Korea carried out on 13,570 participants aged ≥40 years demonstrated that increasing consumption of green tea from zero to ≥2 times per day decreased the risk of COPD in the population [196].

### 5.5. EGCG in Lung Fibrosis

Lung fibrosis may develop as a diffuse, progressive remodeling of the lung parenchyma with extracellular matrix deposition and irreversible scarring due to unknown reasons, e.g., idiopathic pulmonary fibrosis, or may originate from known reasons such as ARDS-induced fibrosis, chronic hypersensitivity pneumonitis, asbestosis, drug-induced pulmonary fibrosis, etc. [197,198].

EGCG possesses many favorable properties that can be useful for treatment of lung fibrosis, as well. Lung fibrosis typically develops as a result of chronically persisted inflammation and oxidative stress, tissue remodeling, and repair processes, leading to excessive deposition of connective tissue and destruction of normal lung architecture [34]. These changes result from changes in several pathways including activation of NF-κB and resulting overproduction of pro-inflammatory cytokines (TNFα, IL-1, IL-6, IL-8, etc.) and proteolytic enzymes cleaving extracellular matrix such as MMP or adamalysins, depletion of antioxidant system Nrf2, activation of growth factors, increased expression of fibrogenic and angiogenic factors resulting into elevated production of MMPs, smooth muscle actin (SMA), collagen, etc. [199,200].

The effect of EGCG was tested in various animal models of lung fibrosis (Table 3). For instance, in a bleomycin-induced model of lung fibrosis characterized by initial inflammation and secondary fibrosis, administration of EGCG prevented a decrease in body weight, elevated levels of both enzymic antioxidants (SOD, catalase, GPx, and GR) and nonenzymic antioxidants (reduced GSH and vitamins C, E, and A), reduced lung edema expressed as a wet–dry lung weight ratio, decreased content of hydroxyproline, a collagen breakdown product, and markers of lipid peroxidation, and improved the histological picture of the lung [188]. Additional results from this model showed that EGCG prevented a bleomycin-induced increase in generation of ROS, restored a decrease in antioxidant status, and enhanced Nrf2 activity. EGCG also reduced markers of inflammation such as levels of NF-κB, TNFα, IL-1β and MPO activity and mitigated histological signs of inflammation and lung injury [34]. In addition, EGCG decreased levels of hydroxyproline and glycoconjugates, metabolic products of collagen, reduced matrix degrading lysosomal hydrolases, and improved ultrastructural changes in the lung [34,35]. More recent experiments performed by these authors on the rat model of fibrosis showed that EGCG decreased levels of MMP-2 and MMP-9, lowered expression of TGF-β1, Smads, and α-SMA, and the mentioned anti-fibrotic effects were also validated in vitro [189]. Attenuation of TGF-β1 signaling and activation of MMP-dependent collagen I turnover by EGCG has recently been demonstrated in cultured precision-cut lung slices from explants of patients with idiopathic pulmonary fibrosis undergoing transplantation [201].

Favorable effects of EGCG have also been shown in other animal models of lung fibrosis. In irradiation-induced fibrosis, EGCG reduced mortality, improved lung histological changes, decreased serum levels of TGF-β1, IL-6, IL-10, and TNFα, and reduced collagen deposition and (myo)fibroblast proliferation [106]. In addition, EGCG prevented oxidative stress, as expressed by activated Nrf2 and associated antioxidant enzymes HO-1 and NAD(P)H:quinone oxidoreductase-1 (NQO-1), enhanced activity of other antioxidant SOD, and decreased levels of MDA in the lungs, a marker of lipid peroxidation [106]. In cyclophosphamide-induced pulmonary fibrosis in rats, pretreatment with a green tea extract prevented inflammatory, oxidant, and fibrotic changes compared to the nontreated control [114]. Similarly, a green tea extract ameliorated paraquat-induced pulmonary fibrosis by suppression of oxidative stress and decrease in endothelin (ET)-1 expression [190].

Another mechanism contributing to the pathophysiology of pulmonary fibrosis is upregulation of heat shock protein (HSP)47. HSP47, a collagen-specific molecular chaperone, regulates procollagen production in the endoplasmic reticulum; thus, HSP47 is essential for proper collagen synthesis and secretion [202]. HSP47 expression was increased in type II pneumocytes, myofibroblasts, and macrophages of bleomycin-induced pulmonary fibrosis models [203,204] as well as in patients with idiopathic pulmonary fibrosis [205]. EGCG has recently been identified as a potent inhibitor of HSP47; thus, its therapeutic effects are partially mediated also through this mechanism [206].

### 5.6. EGCG in Lung Silicosis

A positive effect of EGCG can be also expected in lung silicosis, where the chronic inflammation and fibrotizing processes are closely related to massive and long-lasting oxidative stress evoked by persistence of inhaled silica particles in the lung, usually as a result of the occupational exposure [19] (Table 3). In a recently published study, delivery of EGCG, but especially of EGCG-encapsulated poly(butyl-2-cyanoacrylate) nanoparticles, to rats with lung silicosis alleviated the lung fibrosis including accumulation of collagen and production of α-SMA, and restored a decrease in body weight of silica-injured animals [191]. Similarly, in our pilot experiments in silica-injured rats, administration of EGCG (20 mg/kg i.p.) decreased percentage of inflammatory cells in BALF, and reduced accumulation of collagen and smooth muscle mass in the bronchioles and pulmonary vessels [207].

### 5.7. Lung Cancer

Lung cancer is one of the most frequent types of cancer occurring in the adult population. Lung cancer may be triggered by abundant concentrations of ROS [75] generated due to inhalation of cigarette smoke or exposure to other carcinogens such as indoor cooking with wood/biomass, occupational exposure to various carcinogens including asbestos, etc., or due to cell dysplasia and tissue remodeling in chronic inflammatory diseases (bronchial asthma, COPD, tuberculosis, etc.), which progress to carcinogenesis [208]. A shift in oxidant/antioxidant balance activates several signaling pathways that induce DNA damage and mutagenesis and enhance cell proliferation [70]. On the other hand, these cancerogenic changes are limited by anticancer mechanisms covering cell cycle arrest and cell death via processes of apoptosis, autophagy, and necroptosis [70,209]. 

Among lung cancers, non-small cell lung carcinoma (NSCLC) represents about 80%, and because of late detection in a majority of patients, this disease has a poor prognosis [210]. The process of lung cancer carcinogenesis is activated by receptor tyrosine kinases such as EGFR and c-Met (also known as hepatocyte growth factor receptor). NSCLC cells overexpress EGFR and c-Met, which recruit downstream signaling molecules such as extracellular signal-regulated kinase (ERK)1/2, MAPK, STAT3, protein kinase B (PI3K-Akt) or mammalian target of rapamycin (mTOR), enhancing cell growth and migration [211,212]. EGFR also regulates the Bax/Bcl-2 cascade, inhibiting apoptosis and inducing resistance to chemotherapy. Therapy of NSCLC is specifically targeted to the mentioned receptor tyrosine kinases; however, efficacy of the tyrosine kinase inhibitors may be limited by additional mutations in EGFR and compensatory activations of other pathways [210,212].

For this reason, EGCG appears to be potentially beneficial, as it decreases carcinogenic activity through cessation of receptor kinases EGFR and c-Met, but also platelet-derived growth factor receptor (PDGFR), insulin-like growth factor receptor (IGFR), vascular endothelial growth factor receptor (VEGFR), and it also suppresses the downstream kinases, including Erk1/2, STAT3, and PI3K [213]. Activation of EGFR signaling was inhibited by EGCG in three different NSCLC cell lines, including wild-type EGFR and EGFR with additional mutations, which resulted in the mitigation of cell proliferation and migration in NSCLC cell lines [134]. In another study, EGCG cut off the cell proliferation and activation of not only EGFR, but also c-Met, while the combination of EGCG with the EGFR antagonist erlotinib or c-Met inhibitor SU11274 potentiated the antiproliferative effect [210]. The synergistic effect on the growth of lung cancer cells was also shown for combination of EGCG and NF-κB inhibitor BAY11-7082 (Zhang et al. 2019). Moreover, EGCG may enhance cisplatin sensitivity in NSCLC cells, probably via upregulation of copper transporter (CTR)1 by EGCG-induced increase in ROS generation and upregulation of lncRNA nuclear paraspeckle assembly transcript 1 (NEAT1) [214,215]. These results indicate that EGCG may be a valuable adjunct to the standard anticancer agents. The anti-tumor effect of EGCG in NSCLC may be also potentiated by combination of EGCG with other polyphenols, e.g., curcumin [216], thus enhancing the bioavailability of EGCG, decreasing its methylation [217]. 

In addition, EGCG may disrupt the proliferation of lung cancer cells via induction of cell apoptosis by enhancing the Bax and diminishing Bcl-2 and by triggering G2/M cell cycle arrest [218,219,220].

EGCG also diminishes lung cancer stem cell activity, suppresses cell proliferation, and induces apoptosis via downregulation of the Wnt/β-catenin pathway, which is fundamental for maintaining the stemness of cancer stem cells [221].

Cancer can be promoted by various signaling pathways, which are also activated in inflammation, such as transcription factors NF-κB, AP-1, STAT3, protein kinases such as MAPK or JNK, cell adhesion molecules such as ICAM, or COX-2 [67,68,95]. EGCG causes differential inhibition of NF-κB expression in cancer vs. normal cells, with much lower doses of EGCG needed for cancer cells than for healthy cells to demonstrate the effect, i.e., inhibitory effect on NF-κB is seen predominantly in cancer cells [222]. Suppression of lung cancer cell proliferation, mediated partially by inhibition of NF-κB, may require high doses of EGCG; however, combined administration of EGCG with, e.g., NF-κB inhibitor may exert significant synergistic effect at relatively low concentrations [223]. Effect of EGCG on NF-κB and other pathways including PI3K/Akt/mTOR and MAPK was demonstrated in bronchial epithelial cells exposed to cigarette smoke, a potent inducer of inflammatory response and predisposing factor for carcinogenesis [224]. In another study, EGCG prevented smoking-induced benzopyrene-DNA adduct formation and precancerous lesions of bronchial epithelial cells in rat lungs via downregulation of CYP1A1 expression. CYP1A1 is a target gene involved in a metabolism of aromatic hydrocarbons (such as benzpyrene) to carcinogens, which is overexpressed due to smoke exposure and also in NSCLC cells [192] (Table 3).

Prevention of tumors is also supplied by potent pro-oxidant action of EGCG, as mentioned before. In a culture of human lung cancer H1299 cells and in xenograft tumors, administration of EGCG increased generation of intracellular and mitochondrial ROS, which was associated with oxidative DNA damage and tumor cell apoptosis in a dose-dependent manner [225].

The chemotherapeutic effect of EGCG may be enhanced by encapsulation, which improves the bioavailability and stability of EGCG. In a patient-derived tumor xenograft model, poly(lactic-co-glycolic acid) nanoparticles loaded with EGCG showed more potent antiproliferative activity, stronger induction of apoptosis, and inhibition of NF-κB activation than free EGCG [226]. Similarly, in the study evaluating effects of EGCG and EGCG-nanoemulsion on cultured human lung cancer cells, EGCG-nanoemulsion effectively inhibited lung cancer cell colony formation, migration, and invasion, probably via activated AMP-activated protein kinase (AMPK) signaling pathway [141].

Besides benefits of EGCG for prevention of cancer and mitigation of its development and metastasis, EGCG may also be valuable for prevention of adverse effects of radiotherapy due to lung cancer as recently demonstrated in Phase 2 Clinical Trial (NCT02577393) [227]. 

### 5.8. Pulmonary Hypertension

Pulmonary hypertension (PH) is a heterogenic group of disorders characterized by abnormally high values of pressure in the pulmonary arteries. PH may develop in advanced common diseases, such as COPD and left heart disease, or may result from chronic organized thromboemboli or a primary vasculopathy [23]. The treatment of PH includes medicaments from several pharmacological groups: inhibitors of phosphodiesterase type 5, stimulators of soluble guanylate cyclase, antagonists of endothelin receptor, prostacyclin analogues, and prostacyclin receptor agonists [23,228]. However, EGCG may attenuate hypoxia-induced excessive proliferation of pulmonary artery smooth muscle cells and vascular remodeling, which are the main features of PH. EGCG given to rats with hypoxia-induced PH reduced right ventricular systolic pressure, pulmonary vascular remodeling and right ventricular hypertrophy in a dose-dependent manner and prevented mitochondrial fragmentation and smooth muscle cell proliferation via KLF4/MFN-2/p-Erk signaling pathway [229]. In addition, EGCG may be beneficial because its ability to inhibit MMP-2 and -9 [89,230], which are involved in the regulation of homeostasis of extracellular matrix and vascular remodeling and which overexpression is associated with development of PH [231]. 

### 5.9. Pulmonary Embolism

Pulmonary embolism is a serious acute situation which develops when a blood clot from other parts of the body (usually legs) travels to the lung artery and blocks the perfusion of the related area of the lung. Treatment of pulmonary embolism is complex and besides other approaches includes the use of anticoagulants and fibrinolytic therapy [24]; however, EGCG may also be of benefit. In the study by Kang et al., the effects of green tea catechins and EGCG were studied on the murine model of pulmonary thrombosis and platelet aggregation was evaluated in rats and healthy volunteers. Improved survival was found in a dose-dependent manner, as well as longer bleeding time, in mice, decreased adenosine diphosphate (ADP)- and collagen-induced platelet aggregation was detected in a dose-dependent manner ex vivo in rats, and decreased ADP-, collagen-, epinephrine-, and calcium ionophore A23187-induced platelet aggregation were found for human blood [232], probably via anti-platelet activities of the given agents.

## 6. Advanced EGCG Delivery Forms

As mentioned before, the effectiveness of EGCG is limited due to its poor pharmacokinetics and low bioavailability after oral delivery [60]. After oral intake, EGCG is already enzymatically transformed by the saliva, which hydrolyzes EGCG by esterases [55]. The process continues in the intestine and liver. Due to glucuronidation and sulfation of the hydroxyl groups and O-methylation of the catechol groups through UDP-glucuronosyltransferase, phenolsulfotransferase and catechol-O-methyltransferase, O-methylated and both O-methylated and glucuronidated conjugates are generated, which still have similar biological activity to free EGCG [233]. Cellular uptake is achieved by passive transport, while the affinity to biomembranes is determined by higher hydrophobicity of EGCG compared to other catechins [60]. EGCG undergoes significant degradation by epimerization and auto-oxidation not only in the biological fluids but also during the processing and storage of tea [234]. In auto-oxidation, EGCG loses hydrogen atoms, and potentially damaging substances such as semiquinone radical intermediates, superoxide, and quinone oxidized products are generated [235]. Another process of degradation of EGCG is the epimerization of EGCG to its trans-epimer, which has similar properties to the cis form of EGCG, and no toxic by-products are generated [236]. Epimerization occurs when auto-oxidation is prevented by antioxidants, and this process is reversible [237].

To improve the bioavailability of catechins including EGCG, novel techniques such as nanostructure-based drug delivery system (encapsulation), molecular modification of EGCG, and co-administration of catechins with other bioactive approaches have been tested [62]. For encapsulation, several types of nanovehicle have been used, including gold-, mesoporous silica-, chitosan-, lipid-, carbohydrate- and protein-based nanoparticles [62,238,239]. As precisely reviewed by Li et al., the action of EGCG delivery systems is based on (1) coating with self-polymerized EGCG on the surface of nanoparticles enhancing cellular uptake of the nanovehicles, (2) surface functionalization with specific molecules (chitosan, folic acid, gallic acid, and chlorogenic acid), enhancing stability, cellular uptake, and drug controllable release, (3) targeted molecular modification by peptides or aptamers to target specifically the cell receptors in cancer cells, and (4) preparation of multi-modal therapeutics co-delivery systems with, e.g., chemotherapeutics, therapeutic genes or photo-sensitizers to enable EGCG-involved cancer combination therapy [238]. 

The use of these techniques may effectively enhance the therapeutic effect, as demonstrated in several studies. For instance, EGCG-gold nanoparticles showed more potent anti-tumor activity than conventional gold nanoparticles [240]. Colloidal mesoporous silica-based nanoparticles prolonged the half-life of EGCG and enhanced the therapeutic effect of EGCG, elevating hydrogen peroxide production [241]. EGCG loaded with solid lipid nanoparticles caused cytotoxicity against cancer cells that was several times higher [242]. Recently, glyceryl monooleate (GMO)-based nanoparticles utilizing encapsulation of EGCG inside monoolein nanoparticles were tested in human lung carcinoma cells and exerted more than additive cytotoxic activity to the carcinoma cells [239]. The use of EGCG-loaded chitosan-gellan gum bipolymeric nanohydrogels resulted in sustained drug release and enhanced antibacterial and antioxidant activity [243]. In addition to the mentioned types of nanoparticles given individually, several combinations of the delivery systems have been tested to upgrade the therapeutic effect. For instance, glycosylated ferritin-chitosan nanoparticles effectively protected EGCG from pepsin and trypsin digestion and improved absorption of EGCG [244].

Another important approach is the structural modification of EGCG. Insertion of a specific chain of chemical groups into the molecule of EGCG should protect the reactive hydroxyl groups of EGCG and thereby increase the stability, improve the interaction of EGCG with lipid membranes, and to enhance cellular absorption [245]. Favorable results have been obtained from methyl-protected EGCG [246], EGCG monoester derivatives [247,248], alkyl-analogues [245,249], and glycoconjugates [250,251].

The third possibility for enhancing catechin bioavailability is co-administration with other bioactive substances such as ascorbic acid [252].

## 7. Adverse Effects and Drug Interactions of EGCG

As mentioned before, the antioxidant effects of EGCG are concentration dependent and related to scavenging free radicals and chelating metal ions to prevent generation of ROS. However, EGCG undergoes auto-oxidation and induces production of ROS in mitochondria and cell apoptosis, which may lead to inhibition of cancer, while simultaneously inducing expression of genes related to antioxidant defense [253,254]. Thus, while at moderate levels of EGCG, Nrf2-mediated production of ROS may be beneficial [255], high doses of EGCG may lead to cellular damage and side effects [256]. However, as the low doses may have lower effectiveness, an equilibrium between a necessary therapeutic dose and a risk of side effects due to over-dosing should be carefully considered [256]. High doses of green tea catechins (>600 mg/day) increase a liver enzyme activity [257,258]; therefore, to reduce a risk of hepatotoxicity, the tolerable upper intake of tea catechins has been set in some European countries (e.g., 300 mg of green tea catechins per day in Italy and France) [259]. However, for stronger therapeutic effects, e.g., in cancer, higher doses (600–900 mg or more) of catechins may be needed [256,260]. This problem may be solved by introduction of novel ways of administration of green tea catechins, such as encapsulation (see further in the text), which may increase the effectiveness and possibly decrease adverse effects because of avoiding direct contact with biological barriers and enzymes.

The Minnesota Green Tea Trial showed that about 5% of post-menopausal women who were treated daily for 12 months by high oral dose of green tea extract containing 843 mg of EGCG had increased serum levels of alanine aminotransferase or aspartate aminotransferase [261] and reported higher incidence of nausea and dermatologic adverse effects [262]. Higher sensitivity to EGCG and increased EGCG-associated risk of hepatotoxicity may be linked with genetic background [263]. Predisposed people with some genetic polymorphisms, e.g., with low activity of catechol-O-methyltransferase gene catalyzing the methylation of the phenolic groups at the 4- or 4′- position of EGCG, may be more susceptible for EGCG toxicity [256,264]. However, these associations have not yet been elucidated sufficiently.

As an interesting topic to be investigated in future, the hypothesis that the daily amount of tea catechins taken in tea beverages continuously through the day could be less toxic than the same amount of catechins in isolated forms (or pure EGCG) given as a bolus arose. In addition, catechin toxicity may be decreased by a protective action of caffeine and theanine present in tea; however, this concept needs to be confirmed experimentally [256].

On the other hand, some factors may increase the toxicity of EGCG. For instance, co-administration of EGCG and diethyldithiocarbamate, a representant of dithiocarbamates (DTC) and a metabolite of disulfiram, synergistically increased liver toxicity and lethality. DTC increases EGCG oxidation and toxicity in the liver by increasing level of redox-active copper [265] what may be reduced by addition of copper into the diet leading to subsequent up-regulation of ceruloplasmin activity [266]. Similarly, isothiocyanates upon conjugation with GSH can form DTC increasing EGCG toxicity [256]. 

Nevertheless, interactions of EGCG with other antioxidants have not yet been sufficiently studied. Taking high doses of EGCG together with high doses of other polyphenols in the diet could combine their effect, causing liver toxicity by generating excessive amounts of ROS and depleting the oxidant defense system in cells [256]. On the other hand, induction of antioxidant and cytoprotective enzymes, such as Nrf2-dependent cytoprotective enzymes, by pretreatment with a lower dose of antioxidant could reduce the toxicity of subsequent delivery of a high dose of EGCG. This effect was demonstrated after pretreatment with melatonin which prolonged a survival time of mice subsequently treated with EGCG, and reduced EGCG-induced liver injury and hepatic Nrf2 activation [267] as well as after pretreatment with a moderate dose of EGCG, which prevented the hepatotoxicity caused by the subsequently administered high bolus of EGCG [268].

In addition, interaction with EGCG may change the therapeutic effect of certain drugs. For instance, EGCG decreased bioavailability of anti-fibrotic drug nintedanib in patients with pulmonary fibrosis [269], but increased tumor-inhibitory effects of doxorubicin in murine models of tumors [270].

## 8. Conclusions

Results of recent studies indicate possible benefits of EGCG in the treatment of various diseases. Thanks to its anti-inflammatory, antioxidant, anti-fibrotic and anti-remodeling effects and relative safety in lower doses EGCG may serve as an adjuvant agent for treatment or prevention of a variety of acute and chronic respiratory disorders, as well. Nevertheless, further research is needed to find out the appropriate dosing for achieving sufficient therapeutic effects while minimizing adverse effects. In addition, the action of EGCG in individual patients should be studied in association with their genome as some genetic polymorphisms may influence the efficacy and occurrence of side effects in the predisposed patients. Another important challenge for the future is to enhance the therapeutic efficacy of EGCG using new technologies and methods. Progress in this field indicates that more potent, stable, and specific active formulations of catechins with targeted delivery and rapid release of therapeutically appropriate doses may represent promising novel approaches not only for the treatment of cancer, but also for other diseases including the respiratory ones.

## Figures and Tables

**Table 1:** Major targets of action of EGCG in the lung.

Targets	Modulation by EGCG	Biological Effects
Cell surface receptors		
EGFR VEGFR	Inhibition inhibition	inhibited proliferation of lung non-small cancer cells [134] anti-angiogenic action [135]
TLR4	inhibition	anti-inflammatory action [136]
SARS-CoV-2 spike receptor binding domain ACE2	inhibition	inhibition of SARS-CoV-2 from entering into cells [40]
Intracellular signaling pathways		
MAPK	inhibition	anti-inflammatory and anti-tumorous action [39,137]
PI3K/Akt/eNOS	inhibition	vasorelaxation, anti-inflammatory and anti-tumorous action [138]
COX-2	inhibition	anti-inflammatory and anti-tumorous action [139]
Cytosolic calcium	elevation	various biological actions including induction of apoptosis [140]
AMPK	activation	anti-tumorous action [141]
Nuclear transcription factors		
NF-κB	inhibition	anti-inflammatory action, anti-oxidant action, inhibited proliferation of cancer cells [142]
AP-1	inhibition	anti-inflammatory action, inhibition of cell growth [71,143]
Nrf2/HO-1	activation	anti-oxidant action, anti-inflammatory action [106]
STAT1	inhibition	inhibited apoptosis of lung epithelial cells, anti-inflammatory and anti-tumorous action [144,145,146]
STAT3	inhibition	induction of apoptosis and anti-proliferative effect, anti-inflammatory action [147,148]

Abbreviations: ACE2: angiotensin-converting enzyme 2, AMPK: adenosine monophosphate-dependent kinase, AP-1: activator protein 1, COX-2: cyclooxygenase-2, EGCG: epigallocatechin-gallate, EGFR: epidermal growth factor receptor, eNOS: endothelial nitric oxide synthase, HO-1: heme oxygenase-1, MAPK: mitogen-activated protein kinase, NF-κB: nuclear factor kappa-B, Nrf2: nuclear factor erythroid-derived 2-like 2, PI3K/Akt: phosphoinositide-3-kinase-protein kinase B/Akt, SARS-CoV-2: severe acute respiratory syndrome coronavirus 2, STAT1/3: signal transducer and activator of transcription 1/3, TLR4: toll-like receptor 4, VEGFR: vascular endothelial growth factor receptor.

**Table 2:** EGCG in the treatment of acute lung injury and respiratory infections including COVID-19 (animal models). For more details, see the text.

Animal Model	Species	EGCG Dose/Way of Delivery	Major Findings	Study
LPS-induced ALI	BAL/c mice	EGCG 10 mg/kg i.p., given 1 h before LPS (10 mg/kg i.p.)	↓ inflammation, ↓ injury, ↑ gas exchange	[136]
LPS-induced ALI	C57BL/6 mice	EGCG 15 mg/kg i.p., given 1 h before and 3 h after LPS (2 mg/kg i.t.)	↓ inflammation, ↓ oxidation markers, ↓ lung injury and ↑ regeneration capacity	[98]
LPS-induced ALI	BALB/c mice	EGCG 10 mg/kg i.p., given 1 h before LPS (5 mg/kg i.t.)	↓ inflammation, ↓ lung edema, ↓ MPO and PK Cα	[39]
Fluoride-induced ALI	Wistar rats	EGCG (40 mg/kg) administered 90 min before oral fluoride, given for 4 weeks	↓ markers of oxidative stress, ↑ antioxidants, ↓ inflammation	[149]
*Pseudomonas aeruginosa*-induced pneumonia	ICR mice	EGCG 20, 40 or 80 mg/kg i.g., *P. aeruginosa* instillation (2.5 × 10^8^ CFU i.t.)	↓ inflammation, ↓ lung injury, ↓ *P.* *aeruginosa* load and virulence	[41]
*Mycobacterium tuberculosis*-induced pneumonia	BAL/c mice	Encapsulated EGCG (10, 20 and 50 mg) given by inhalation or EGCG (2.5 mg) by oral gavage, given 4 weeks after inoculation (2.8 × 10^6^ CFU/mL i.t.)	↓ inflammation, ↓ bacterial burden	[150]
*Influenza A*-induced pneumonia	BAL/c mice	EGCG (10, 20 or 40 mg/kg/d, p.o.) for 5 d, *influenza A* infection on 3rd day of EGCG	↑ survival, ↓ inflammation, ↓ virus yields, ↓ ROS	[151]
SARS-CoV-2-induced pneumonia	C57BL/6 mice	EGCG 10 mg/kg daily p.o. for 14 days, given after infection with 10 μL of HCoV-OC43 virus (107 PFU/mL) i.n.	↓ viral replication	[152]

Abbreviations: ALI: acute lung injury, CFU: colony forming units, LPS: lipopolysaccharide, i.g.: intragastric administration, i.n.: intranasal administration, i.p.: intraperitoneal administration, i.t.: intratracheal administration, MPO: myeloperoxidase, p.o.: peroral administration, PFU: plaque-forming units, PK Cα: protein kinase Cα, ↓: decrease, ↑: increase.

## Data Availability

Data is contained within the article.
